# Chromosome copy number changes carry prognostic information independent of KIT/PDGFRA point mutations in gastrointestinal stromal tumors

**DOI:** 10.1186/1741-7015-8-26

**Published:** 2010-05-14

**Authors:** Mara Silva, Isabel Veiga, Franclim R Ribeiro, Joana Vieira, Carla Pinto, Manuela Pinheiro, Bárbara Mesquita, Catarina Santos, Marta Soares, José Dinis, Lúcio Santos, Paula Lopes, Mariana Afonso, Carlos Lopes, Manuel R Teixeira

**Affiliations:** 1 Department of Genetics, Portuguese Oncology Institute - Porto, Rua Dr. António Bernardino Almeida, 4200-072 Porto, Portugal; 2 Department of Oncology, Portuguese Oncology Institute - Porto, Rua Dr. António Bernardino Almeida, 4200-072 Porto, Portugal; 3 Department of Surgery, Portuguese Oncology Institute - Porto, Rua Dr. António Bernardino Almeida, 4200-072 Porto, Portugal; 4 Department of Pathology, Portuguese Oncology Institute - Porto, Rua Dr. António Bernardino Almeida, 4200-072 Porto, Portugal; 5 Institute of Biomedical Sciences Abel Salazar (ICBAS), University of Porto, Largo Prof. Abel Salazar, 4099-003 Porto, Portugal

## Abstract

**Background:**

Oncogenic point mutations in *KIT *or *PDGFRA *are recognized as the primary events responsible for the pathogenesis of most gastrointestinal stromal tumors (GIST), but additional genomic alterations are frequent and presumably required for tumor progression. The relative contribution of such alterations for the biology and clinical behavior of GIST, however, remains elusive.

**Methods:**

In the present study, somatic mutations in *KIT *and *PDGFRA *were evaluated by direct sequencing analysis in a consecutive series of 80 GIST patients. For a subset of 29 tumors, comparative genomic hybridization was additionally used to screen for chromosome copy number aberrations. Genotype and genomic findings were cross-tabulated and compared with available clinical and follow-up data.

**Results:**

We report an overall mutation frequency of 87.5%, with 76.25% of the tumors showing alterations in *KIT *and 11.25% in *PDGFRA*. Secondary *KIT *mutations were additionally found in two of four samples obtained after imatinib treatment. Chromosomal imbalances were detected in 25 out of 29 tumors (86%), namely losses at 14q (88% of abnormal cases), 22q (44%), 1p (44%), and 15q (36%), and gains at 1q (16%) and 12q (20%). In addition to clinico-pathological high-risk groups, patients with *KIT *mutations, genomic complexity, genomic gains and deletions at either 1p or 22q showed a significantly shorter disease-free survival. Furthermore, genomic complexity was the best predictor of disease progression in multivariate analysis.

**Conclusions:**

In addition to *KIT/PDGFRA *mutational status, our findings indicate that secondary chromosomal changes contribute significantly to tumor development and progression of GIST and that genomic complexity carries independent prognostic value that complements clinico-pathological and genotype information.

## Background

Gastrointestinal stromal tumors (GIST) represent the most common mesenchymal tumors of the gastrointestinal tract [1]. A diagnosis of GIST involves a multidisciplinary approach that combines clinical, pathological, and genetic features. Mutually exclusive activating mutations in *KIT *or *PDGFRA *occur in 85 to 90% of the cases and are considered primary events in GIST pathogenesis [1-3]. These genes encode type III transmembrane receptor proteins which, upon connection to their respective ligands, activate downstream signaling pathways involved in cell proliferation and survival [4-6].

Whereas oncogenic *KIT *or *PDGFRA *mutations seem vital to promote the neoplastic transformation, additional somatic alterations are presumably necessary for the biological and clinical progression of these tumors and may explain the different responses to targeted therapy seen in these patients. Genome-screening methodologies, such as conventional cytogenetics and comparative genomic hybridization (CGH), have been applied in order to identify these changes. Some chromosomal alterations, such as losses at 1p, 14q, and 22q, are particularly frequent, suggesting the existence of tumor suppressor genes in these regions that could be important in tumor progression [7-10]. Although this cytogenetic fingerprint of GIST has been defined, the target genes involved in these regions remain undiscovered [11]. Furthermore, the relationship between the pattern of *KIT *and *PDGFRA *oncogenic mutations and that of cytogenetic changes has not been systematically studied, precluding a full understanding of the genetic pathways involved in GIST development.

In this work, we assessed the genetic background of a consecutive series of 80 patients diagnosed with GIST. *KIT *or *PDGFRA *mutations were evaluated in all samples using direct sequencing analysis. For a subset of 29 patients with fresh-frozen tisue, CGH was used to screen for chromosomal copy number aberrations. Cytogenetic and molecular genetic findings were integrated and correlated with clinico-pathological parameters, including imatinib/sunitinib therapy response.

## Methods

### Clinical samples

A series of 80 patients diagnosed with GIST and submitted to surgery with curative intent were included in this study. The majority of patients was diagnosed and treated at the Portuguese Oncology Institute - Porto, with the exception of six cases that were provided by other institutions. Patients had received no treatment prior to surgery. Fresh-frozen tumor samples from 29 patients were available for mutational and CGH analyses, whereas for the remaining cases mutational analyses were performed in formalin-fixed, paraffin-embedded tissue sections. In all cases, hematoxylin and eosin stained sections from representative tissue blocks were reviewed by expert pathologists to confirm a diagnosis of GIST and to evaluate relevant histopathological parameters. Immunohistochemistry for CD117 followed the standard avidin-biotin-peroxidase complex method with a commercial polyclonal antibody at a 1/600 dilution (A4502, Dako, Glostrup, Denmark). Other clinical and demographic variables, such as age at diagnosis, gender, tumor size, and tumor location (divided into stomach, small intestine, rectum and colon, and outside the GI tract), were obtained Additional file 1. Patients with tumors that eventually recurred or that developed metastatic lesions (n = 36) were treated with imatinib in accordance with the guidelines followed at the IPO-Porto. Second-line therapy for patients that progressed or were intolerant to imatinib (n = 2) was sunitinib. This study was approved by the institutional review board and was performed in accordance with national regulations (law 12/2005).

### KIT and PDGFRA mutation screening

DNA isolation from formalin-fixed, paraffin-embedded tumor samples and from physically disaggregated fresh-frozen tissue fragments was performed using an adaptation of the technique described by Lungu and colleagues [12] or a salting-out-chloroform mixed methodology [13], respectively. Using the DNA extracted from each sample, *KIT *(exons 9, 11, 13, and 17) and *PDGFRA *(exons 12, 14, and 18) target sequences were amplified by polymerase chain reaction (PCR) on a standard termocycler. Primers and conditions were as described in the literature [14,15]. Direct sequencing was performed on an ABI PRISM 310 automatic sequencer using the Big Dye Terminator Chemistry (Applied Biosystems, Foster City, CA, USA), according to the manufacturer's recommendations. All results were confirmed with a second independent analysis.

### Comparative genomic hybridization

Fresh-frozen tumor samples from 29 patients were analyzed by CGH following the procedure of Kallioniemi *et al*. [16], with modifications previously described [17]. Samples were analyzed with a Cohu 4900 CCD camera using an automated filter wheel coupled to a Zeiss Axioplan fluorescence microscope (Zeiss, Oberkochen, Germany) and a Citovysion system version 3.9 (Applied Imaging, Santa Clara, CA, USA). For each sample, data from 10 cells were combined to generate average ratio profiles with 99% confidence intervals and aberrations were scored whenever the sample profile and the standard reference profile at 99% did not overlap [18]. Description of the CGH copy number changes followed the guidelines suggested by the International System for Human Cytogenetic Nomenclature (ISCN) 2005 [19].

### Statistical analysis

Relevant clinico-pathological (gender, age, tumor size, tumor location and patient risk groups) and genetic (mutation status and chromosomal imbalances) variables were cross-tabulated and analyzed using the chi-square or Fisher's exact test. The number of chromosomal aberrations was compared within groups of samples with different mutation genotypes using the non-parametric Mann-Whitney U test. Kaplan-Meyer survival curves using log-rank test were computed for relevant clinical and genetic events. For statistical purposes, patients with recurrent or metastatic lesions were included in the high-risk group. A *P*-value lower than 0.05 was considered statistically significant. All analysis was performed using the Statistical Package for Social Sciences (SPSS) software, version 15 (SPSS Inc., Chicago, IL, USA).

## Results

### Clinicopathologic characteristics of the patients

A total of 80 patients diagnosed with GIST were enrolled in this study. Tumor location was obtainable in 79 cases, of which 67 corresponded to primary lesions Additional file 1. Twelve recurrent or metastatic lesions were analyzed due to lack of the primary sample. For four of the patients, a second sample collected after disease progression could additionally be assessed, increasing the number of lesions submitted to sequencing analysis to 84. Tumor size was recorded in 70 cases and varied from 1.2 to 45 cm (average 8.8 cm). From the available morphology data, the series included 52 spindle cell tumors, 6 epithelioid lesions, and 13 mixed tumors. Based on the National Comprehensive Cancer Network taskforce guidelines for GIST risk assessment [20], most tumors in this series could be classified as either low/very low risk (n = 27), moderate risk (n = 8), or high risk (n = 27). Expression of the KIT protein (CD117) was assessed in 74 cases. A total of 70 lesions (94.6%) showed a positive staining pattern, whereas two cases were negative and two cases presented inconclusive findings.

### *KIT *and *PDGFRA *mutations

**Figure 1:**
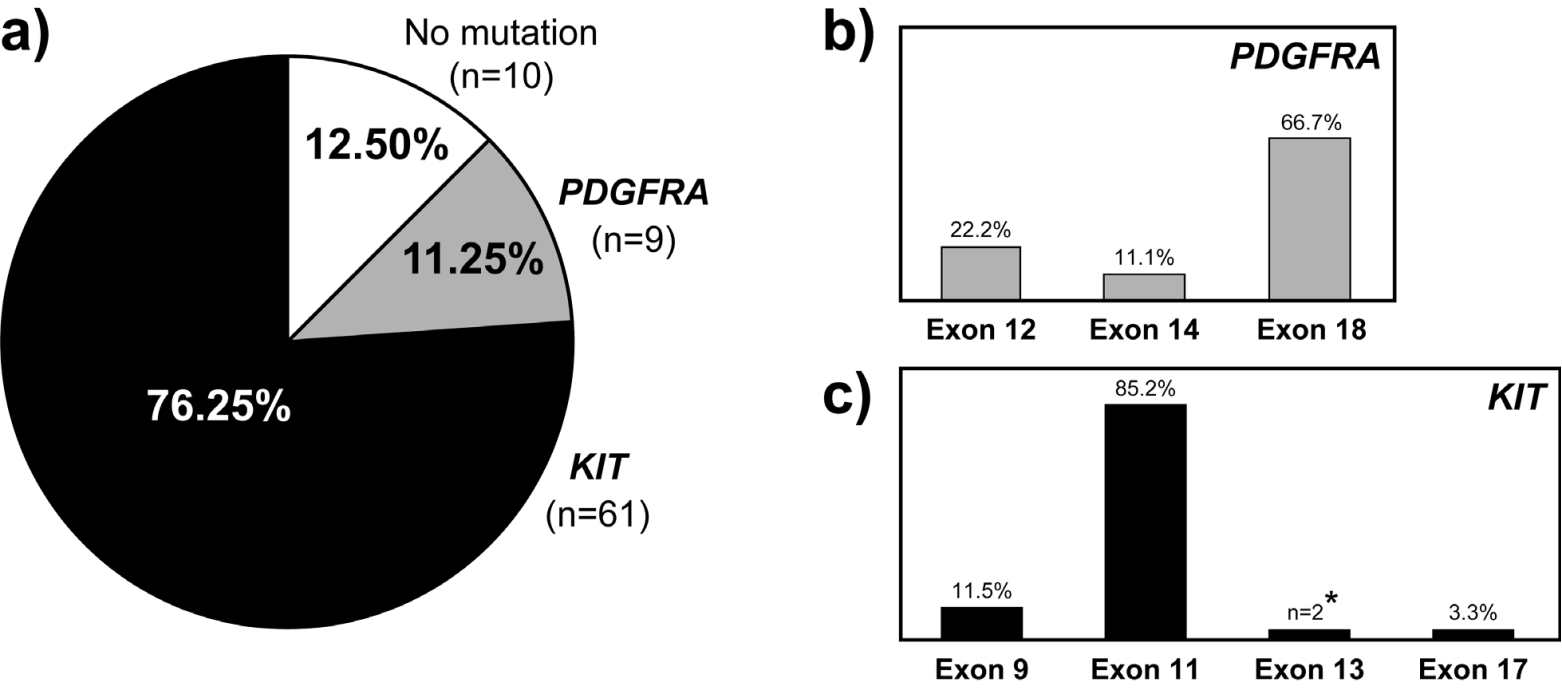
**Mutation profile of *KIT *and *PDGFRA *in 80 GIST**. **a) **mutation frequency per gene; **b) **mutation frequency per *PDGFRA *exon; **c) **mutation frequency per *KIT *exon. Two patients with primary mutations in *KIT *exon 11 acquired the same secondary mutation in *KIT *exon 13 after imatinib treatment. The two patients with KIT exon 17 mutation belong to a family with hereditary GIST, with the mutation being found also in the germline.

Samples from all 80 patients were screened for mutations within exons 9, 11, 13, and 17 of the oncogene *KIT*. Mutations were detected in 61 tumors (76.25%, Figure 1), namely in exon 11 (n = 52), exon 9 (n = 7) and exon 17 (n = 2). The two patients with *KIT *exon 17 mutation were subsequently found to be relatives and the mutation shown to be present in the germline [21]. No primary mutations were found in exon 13. All *KIT *negative cases (n = 19) were then analyzed for mutations in exons 12, 14, and 18 of *PDGFRA*. A total of nine samples (11.25%) showed mutations in this gene, namely in exon 18 (n = 6), exon 12 (n = 2), and exon 14 (n = 1). CD117 staining was seen in six out of eight *PDGFRA *positive cases. The overall mutation frequency for both genes in this series was 87.5% (70 out of 80 tumors). Of note, two tumors with *KIT *exon 11 primary mutations and with an initial response to imatinib, acquired resistance and developed peritoneal or hepatic metastases that presented the same secondary mutation (*KIT *exon 13, p.Val654Glu). A complete description of the mutations and relevant clinical parameters for each patient are detailed in Additional file 1.

### Chromosome copy number changes

**Figure 2:**
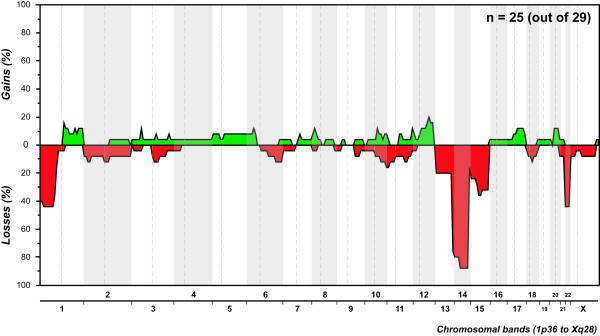
**Copy number profile of patients diagnosed with GIST**. Gains and losses of genetic material are depicted along all chromosomes (X axis).

Out of the 29 GIST submitted to whole-genome screening, 25 (86%) displayed copy number changes (Figure 2, Additional file 2). Most abnormal samples displayed non-complex profiles, with a median of three aberrations per tumor (ranging from one to 28 changes), and losses were 1.5 times more frequent than gains. It is noteworthy that complete or partial loss of 14q was seen in 22 samples (88%), being the sole copy number change in four patients. Other frequent changes included losses at 22q (44%), 1p (44%), and 15q (36%) and gains at 1q (16%) and 12q (20%). All 25 cytogenetically abnormal GIST presented at least one of the losses 1p, 14q, or 22q.

### Integrative analysis of molecular and cytogenetic alterations

Based on previous literature findings, samples submitted to CGH analysis were divided according to mutation genotypes to test for possible correlations. Genomic results were compared between samples with *KIT *exon 9 mutations (n = 3), *KIT *exon 11 deletions/delins (n = 8) or samples with no detectable mutations (n = 1), totaling 12 cases associated in the literature with bad prognosis, versus samples with *KIT *or *PDGFRA *mutations not previously associated with a worse prognosis (n = 13). Strikingly, the former group showed significantly more copy number changes than the latter (median of 6.5 versus 2 aberrations per tumor, *P *= 0.026, Mann-Whitney U test). The three cases with *KIT *exon 9 mutations showed the most complex CGH profiles (median of nine aberrations per tumor), followed by those with exon 11 deletions/delins (median of four aberrations per tumor). It is noteworthy that three of the four cases without detectable CGH alterations showed no mutations in either *KIT *or *PDGFRA*. No significant associations were observed between specific copy number changes and different mutation subgroups. Tumors with *PDGFRA *mutations showed the same overall pattern of alterations seen in those with *KIT *mutations, even if genomic complexity was much lower in the former (median of 2 vs. 5.5 alterations per tumor, respectively, *P *= 0.050, Mann-Whitney U test).

### Therapeutic correlations and survival data

Follow-up data was available in 74 cases (median of 30 months, ranging from 8 to 123 months). During this period, 26 patients (35%) showed disease progression and were subsequently treated with imatinib. According with the most recent clinical records, six of these patients died from their cancer. Most of the samples from the progression group showed *KIT *mutations, namely in exon 11 (n = 21) and exon 9 (n = 3), with only two patients showing no mutations in either gene. The 48 patients that received no adjuvant therapy are currently alive without evidence of disease, with the exception of three non disease-related deaths. Within this group, 41 tumors harbored mutations, namely in *KIT *exon 11 (n = 28), *KIT *exon 9 (n = 4), *PDGFRA *exon 12 (n = 2), *PDGFRA *exon 14 (n = 1), and *PDGFRA *exon 18 (n = 6).

**Figure 3:**
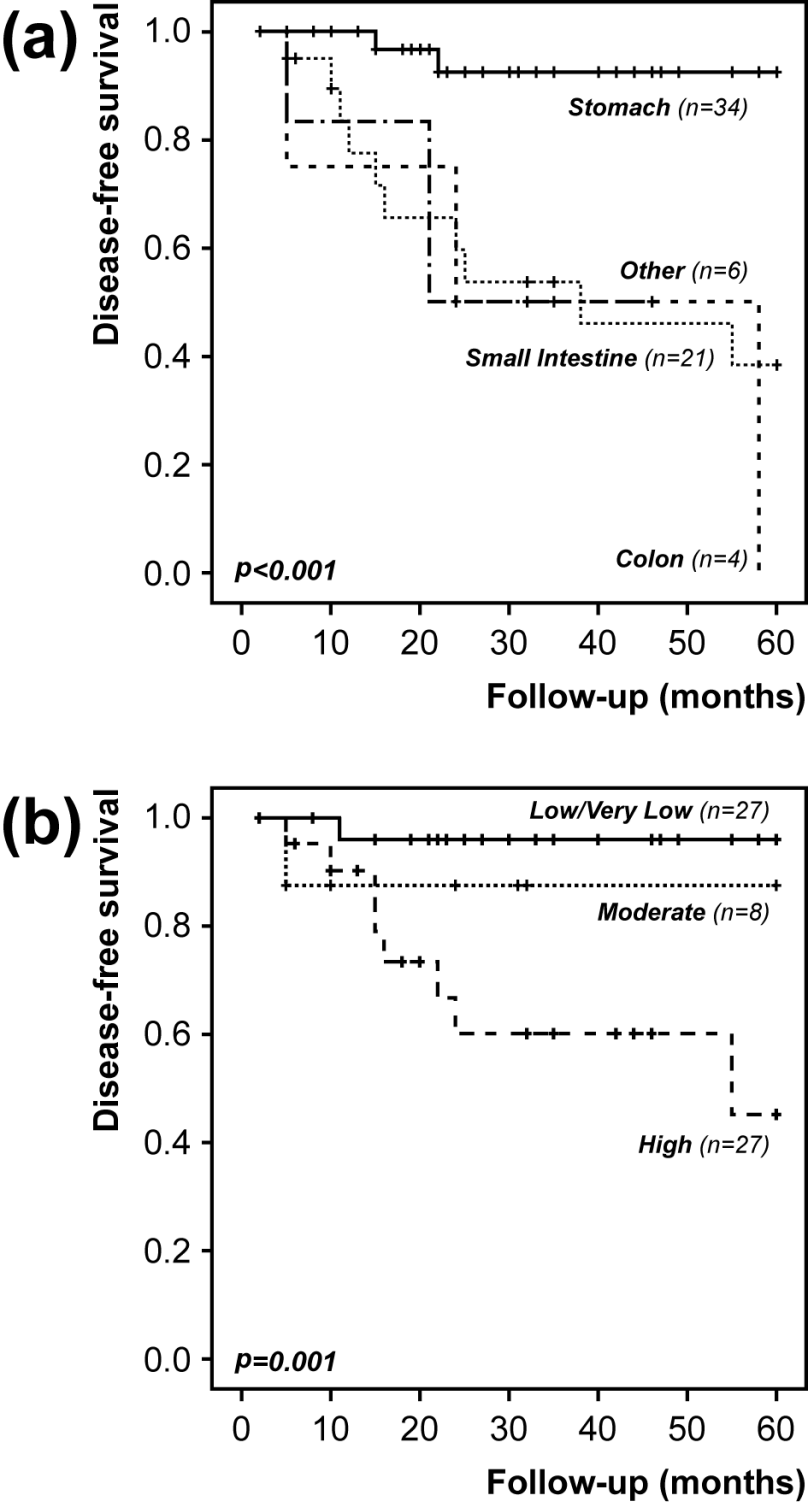
**Five-year disease-free survival curves based on selected clinical variables**. **a) **Tumor location; **b) **Risk groups.

**Figure 4:**
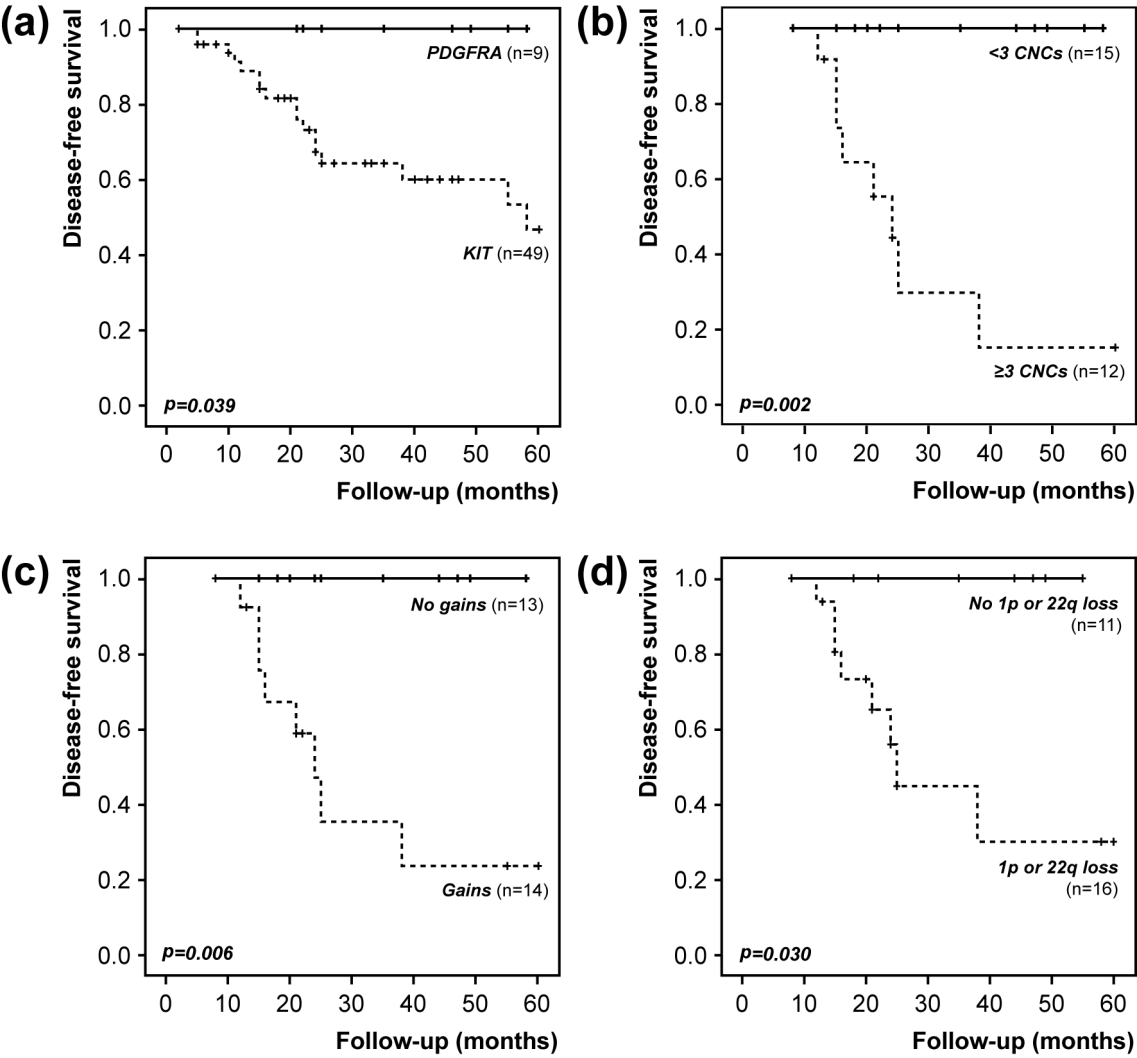
**Five-year disease-free survival based on selected genetic variables**. a) Mutated gene; **b) **genomic complexity; **c) **genomic gains; **d) **1p or 22q deletions.

Disease-specific survival curves were uninformative due to the reduced number of death-from-disease events, and five-year disease-free survival curves were thus computed (Figures 3 and 4, Additional file 3). Stratification according to tumor location showed that lesions in the stomach progressed much less frequently than those in other locations (*P *< 0.001, Figure 3a). Regarding risk groups, most progression events were seen in lesions categorized as high risk (*P *= 0.001, Figure 3b). When patients were categorized based on genetic variables, a more aggressive outcome was seen in patients with *KIT *mutations compared to those with *PDGFRA *mutations (*P *= 0.039, Figure 4a). Based on previous literature reports, patients were additionally categorized according to specific mutations associated with worse prognosis. Patients with *KIT *exon 9 or *KIT *exon 11 deletions/delins (n = 25 cases) showed a tendentiously worse progression-free survival (*P *= 0.091) than those showing mutations in *PDGFRA *or other mutations in *KIT *(n = 33 cases). Within the subgroup of patients with *KIT *exon 11 mutations, the number of progression events in tumors with deletions/delins was significantly higher than those with other mutations (*P *= 0.003, Fisher's test). In multivariate analysis including risk groups and genotype (*KIT *vs *PDGFRA *mutations), a high risk at diagnosis was the strongest predictor of relapse (HR = 9.8, *P *= 0.003, 95% CI = 2.2 to 43.1) (Additional file 4: Supplementary Table 4a)

In the subgroup of patients with CGH data and complete follow-up information (n = 27), genomic complexity was very strongly associated with a worse outcome (*P *= 0.002, Figure 4b). The presence of genomic gains (*P *= 0.006) or deletions at 1p or 22q (*P *= 0.030) were also significantly associated with a shorter progression-free period (Figure 4c and 4d, Additional file 3). Multivariate survival analysis in this subset (using risk groups, genotype status, genomic gains and losses at 1p or 22q) showed that the best predictor of progression was genomic complexity (HR = 13.7, *P *= 0.014, 95% CI = 1.7 to 111.1, Additional file 4: Supplementary Table 4b).

## Discussion

Recent years have seen important breakthroughs that resulted in better diagnostic, prognostic and therapeutic tools for patients with GIST. Identification of a distinctive molecular signature involving *KIT *or *PDGFRA *mutations allowed targeted therapies in patients with metastatic disease, who did not have effective therapeutic options until then. However, additional genomic changes are known to occur in GIST that might influence therapy response and tumor aggressiveness. In the current work, we characterized the mutation profile of *KIT *and *PDFGRA *in a consecutive series of GIST diagnosed and followed at our institution. Gross genomic aberrations were additionally assessed in a subset of these patients, in order to determine the relative contribution of primary and secondary genetic events in GIST as prognostic or/and predictive factors.

Tumor size and mitotic index have been considered the most important prognostic indicators in GIST [22,23]. However, it has been shown that even small GIST can behave aggressively and develop metastases. Indeed, one patient with an intestinal lesion with less than 2 cm and low mitotic rate (patient 20, categorized in the low-risk group) developed metastases and died from the disease 11 months after diagnosis. More recently, anatomic location was also considered of relevance and included in the determination of the risk of recurrence and progression [21,24]. Our findings strongly support this prediction model, as a significant proportion of small intestine or colon GIST developed metastasis, whereas most tumors located in the stomach showed no progression events.

*KIT *and *PDGFRA *activating mutations are mutually exclusive events in GIST that promote the constitutive activation of the receptors and the downstream signaling pathways, resulting in aberrant cell proliferation and apoptosis [2]. The overall frequency of *KIT *and *PDGFRA *mutations in GIST varies in different studies, but is usually higher than 80% [25]. In our 80 samples, we obtained a mutation frequency of 87.2%, with 75.7% of the cases harboring *KIT *mutations and 11.5% showing *PDGFRA *mutations, which is significantly higher than the 63% recently found in a second Portuguese series of GIST [26] and that of another Iberian Peninsula series [27]. It has been suggested that the type and molecular location of different mutational events in GIST carry distinct biological and clinical implications [1,28]. Mutations in the KIT extracellular regulatory domain, coded by exon 9, seem to mimic the conformational changes that follow stem-cell factor (SCF) ligation. The most common mutation found within this location (p.Ala502_Tyr503dup) corresponds to an insertion of six nucleotides [29], and indeed all our tumors with exon 9 mutations displayed this hot-spot alteration. The major mutational hotspot in *KIT *is in exon 11, which encodes the juxtamembrane intracellular domain responsible for modulating KIT enzymatic activity [24]. *KIT *exon 11 deletions have been linked to an aggressive behavior comparing missense and insertion mutations [27,28,30,31]. In our series, 26 out of 52 mutations in this domain corresponded to deletions/delins. Interestingly, 15 of these 26 patients showed disease progression, whereas only four patients in the group with insertions, duplications or missense mutations showed disease progression (*P *= 0.003). It is noteworthy that in two patients with primary *KIT *exon 11 mutations treated with imatinib and in whom additional metastases developed, the same secondary mutation in *KIT *exon 13 (p.Val654Glu), known to confer imatinib resistance [32], was detected. Finally, the two patients in which we identified the same *KIT *exon 17 mutation were recently found to be relatives. In fact, we were able to show that the p.Asp820Tyr was present also in the germline, representing the third example in the literature of hereditary GIST caused by this same mutation [21].

GIST harboring *PDGFRA *mutations share many clinical features with *KIT *mutated tumors, but are mainly gastric and present weak or negative CD117 staining [33]. Existent evidence suggests that *PDGFRA *mutated tumors might be less aggressive [34]. Of the nine cases with *PDGFRA *mutations in our series, three showed weak or only focal CD117 staining, seven were located in the stomach, and all patients are currently alive with no evidence of disease. Interestingly, the hotspot p.Asp842Val mutation, which has been associated with primary resistance to imatinib [3], was detected in four of these cases. However, these four patients were classified in the low risk group and showed no signs of progression thus far. As such, they have not been submitted to imatinib treatment.

In addition to the primary mutation events activating *KIT *or *PDGFRA*, cytogenetic studies have shown additional changes associated with GIST progression [7,35,36]. However, few studies so far have performed genotype and genome analysis in the same samples, preventing a reliable assessment of correlations between primary and secondary genetic events, or their combined prognostic/predictive value [37-39]. In our work, 86% of the GIST submitted to CGH analysis displayed copy number changes. Complete or partial deletions of chromosome 14 were seen in 88% of the abnormal cases, and in four patients this was the sole chromosomal change detected. Additional recurrent cytogenetic aberrations included losses at 22q, 1p, and 15q, as well as gains at 1q and 12q. Genomic complexity (three or more aberrations per tumor), the presence of gains, deletions at 1p, and deletions at 22q were associated with a shorter disease-free survival in this subset of patients, with multivariate analysis evidencing genomic complexity as the best predictor of disease relapse. Strikingly, tumors harboring *KIT *mutations associated with a bad prognosis showed significantly more chromosome copy number changes than those without such mutations. On the opposite side, tumors with *PDGFRA *mutations showed the same overall pattern of alterations seen in those with *KIT *mutations, but the complexity was much lower and no progression events were observed.

## Conclusions

Taken together, our findings suggest that secondary chromosome changes have independent prognostic value in GIST. Furthermore, chromosome level information might also be useful for differential diagnosis, as the pattern of genomic losses of 1p, 14q, and/or 22q is rather characteristic. Additional integrative molecular and cytogenetic studies are necessary to assess the combined diagnostic, prognostic, and predictive value of the several recurrent genetic features of GIST.

## Abbreviations

CCD: charge-coupled device; CGH: comparative genomic hybridization; GI: gastrointestinal; GIST: gastrointestinal stromal tumors; HR: hazard ratio; ISCN: international system for cytogenetic nomenclature; PCR: polymerase chain reaction; SCF: stem cell factor; SPSS: statistical package for social sciences.

## Competing interests

The authors declare that they have no competing interests.

## Authors' contributions

MSi, IV, CP, MP, BM and CS carried out DNA extractions and genotype analysis and interpretations. MSi and JV performed molecular cytogenetic analysis. FRR carried out statistical analysis and drafted the manuscript together with MSi. MSo, JD and LS provided patient clinical data. PL, MA and CL carried out pathological assessment of the tumors. MRT designed and coordinated the study, assisted with scoring the molecular cytogenetic changes, and contributed to manuscript writing. All authors read and approved the final manuscript.

## Pre-publication history

The pre-publication history for this paper can be accessed here:

http://www.biomedcentral.com/1741-7015/8/26/prepub

## Supplementary Material

Additional file 1 **Clinicopathologic characteristics and genotypic results of 80 patients diagnosed with GIST**. Table summarizing clinical and genotypic variables of the 80 patients enrolled in this study. Click here for file

Additional file 2 **Chromosomal imbalances and molecular alterations detected in 29 GIST submitted to CGH analysis**. Table integrating CGH and genotypic data for 29 patients for which both information was available. Click here for file

Additional file 3 **Disease-free survival results for selected variables**. Univariate disease-free survival results for selected genetic and clinical variables, using the Kaplan-Meyer test with Log-rank statistics at 60 months follow-up. Click here for file

Additional file 4 **Multivariate disease-free survival results**. Results of the cox-regression models (forward conditional setting) using the variables that showed significant prognostic value in univariate testing. Click here for file
