# Supplementary material for: Chromosome copy number changes carry prognostic information independent of KIT/PDGFRA point mutations in gastrointestinal stromal tumors
Source: BMC Med. 2010 May 14;8:26. doi: 10.1186/1741-7015-8-26 (PMC2876987; doi:10.1186/1741-7015-8-26)
Supplement: Additional file 1 — Clinicopathologic characteristics and genotypic results of 80 patients diagnosed with GIST. Table summarizing clinical and genotypic variables of the 80 patients enrolled in this study. [file 1741-7015-8-26-S1.PDF]

Supplementary Table 1: Clinicopathologic characteristics and genotypic results of 80 patients diagnosed with GIST

Case ID	Age	Location	Sample type	Risk Group[1]	Status	Gene	Exon	Mutation description[2]	Type	CGH?
2	59	Small intestine	Primary	High	ANED	*KIT*	9	p.Ala502_Tyr503dup	duplication	Yes
12	53	Small intestine	Primary	High	AWD	*KIT*	9	p.Ala502_Tyr503dup	duplication	
18a	61	Small intestine	Recurrence		AWD	*KIT*	9	p.Ala502_Tyr503dup	duplication	Yes
18b	61	Peritoneum	Metastasis		AWD	*KIT*	9	p.Ala502_Tyr503dup	duplication	
23	76	Stomach	Primary	Very low	ANED	*KIT*	9	p.Ala502_Tyr503dup	duplication	
55	63	Small intestine	Primary	High	AWD	*KIT*	9	p.Ala502_Tyr503dup	duplication	Yes
68	24	Stomach	Primary	Low	ANED	*KIT*	9	p.Ala502_Tyr503dup	duplication	
74	73	Small intestine	Primary	High	ANED	*KIT*	9	p.Ala502_Tyr503dup	duplication	
3	42	Peritoneum	Metastasis		DFD	*KIT*	11	p.Trp557_Lys558del	deletion	Yes
4	65	Stomach	Primary	High	ANED	*KIT*	11	p.Lys550_Gln556del	deletion	Yes
19	73	Colon	Primary	High	DFD	*KIT*	11	p.Tyr570_Leu576del	deletion	
24	65	Peritoneum	Primary	High	AWD	*KIT*	11	p.Asp579del	deletion	
28	73	Small intestine	Primary	Moderate	ANED	*KIT*	11	p.Trp557_Glu561del	deletion	
35	77	Stomach	Primary	Low	ANED	*KIT*	11	p.Asp579del	deletion	
36	46	Peritoneum	Primary	High	ANED	*KIT*	11	p.Val560del	deletion	
44	52	Stomach	Primary	Low	ANED	*KIT*	11	p.Val559_Glu561del	deletion	Yes
47	61	Colon	Recurrence		DWD	*KIT*	11	p.Glu554_Val559del	deletion	

Case ID	Age	Location	Sample type	Risk Group[1]	Status	Gene	Exon	Mutation description[2]	Type	CGH?
48	37	Small intestine	Primary	High	AWD	*KIT*	11	p. Val559_Asp572del	deletion	
51	74	Small intestine	Primary	nd	AWD	*KIT*	11	p.Glu554_Val561del	deletion	
52	57	Peritoneum	Metastasis		AWD	*KIT*	11	p.Pro551_Glu554del	deletion	
60	71	Stomach	Primary	High	ANED	*KIT*	11	p.Asp579del	deletion	Yes
62	62	Stomach	Primary	Very low	ANED	*KIT*	11	p.Lys550_Lys558del	deletion	Yes
69	60	Small intestine	Primary	High	ANED	*KIT*	11	p.Met552_Trp557del	deletion	
71	51	Small intestine	Primary	High	AWD	*KIT*	11	p.Lys550_Glu554del	deletion	
11	70	Peritoneum	Metastasis		ANED	*KIT*	11	p.Asn567_Tyr578delinsGluAsp	delins	
13	34	Stomach	Primary	High	ANED	*KIT*	11	p.Tyr553_Leu576delinsAsnCysLeuHisLeu TyrSerSerGln	delins	Yes
16	74	Liver	Metastasis		DFD	*KIT*	11	p.Gln556_Val559delinsHis	delins	
20	61	Small intestine	Primary	Low	DFD	*KIT*	11	p.Val559_Ile571delinsVal	delins	
33	59	Small intestine	Primary	High	AWD	*KIT*	11	p.Glu554_Lys558delinsGlu	delins	
34	59	Small intestine	Primary	High	AWD	*KIT*	11	p.Trp557_Val559delinsPhe	delins	Yes
41	69	Stomach	Primary	Very low	ANED	*KIT*	11	p.Pro551_Gln556delinsThr	delins	
43	49	Stomach	Primary	High	AWD	*KIT*	11	p.Gln556_Val559delinsProThrVal	delins	
45	58	Rectum	Primary	Moderate	ANED	*KIT*	11	p.Lys558_Thr574delinsAsnArgSer	delins	Yes
49	38	Small intestine	Primary	High	AWD	*KIT*	11	p.Asn567_Leu576delinsIle	delins	
10	52	Stomach	Primary	Low	ANED	*KIT*	11	p.Asp572_His580dup	duplication	Yes
29	73	Stomach	Primary	Low	ANED	*KIT*	11	p.Leu576_Arg588dup	duplication	

Case ID	Age	Location	Sample type	Risk Group[1]	Status	Gene	Exon	Mutation description[2]	Type	CGH?
37	46	Small intestine	Primary	Moderate	ANED	*KIT*	11	p.Pro573_Tyr578dup	duplication	
57	57	Stomach	Primary	Low	ANED	*KIT*	11	p.Thr574_Asp579dup	duplication	
61	50	Peritoneum	Metastasis		AWD	*KIT*	11	p.Asp572_ Pro573dup	duplication	
70	41	Stomach	Primary	Moderate	ANED	*KIT*	11	p.Ile571_Asp579dup	duplication	
73	43	nd	nd	nd	DFD	*KIT*	11	p.Ile571_Asp579dup	duplication	
72	76	Stomach	Primary	Low	ANED	*KIT*	11	p.Pro585_Arg586insThrThrGlnLeuProTyr AspHisLysTrpGluPhePro	insertion	Yes
5a	84	Small intestine	Recurrence		DFD	*KIT*	11	p.Trp557Arg	missense	Yes
15a	73	Small intestine	Primary	High	AWD	*KIT*	11	p.Trp557Gly	missense	
15b	73	Small intestine	Recurrence		AWD	*KIT*	11	p.Trp557Gly	missense	Yes
26	42	Small intestine	Primary	High	ANED	*KIT*	11	p.Trp557Arg	missense	
27	26	Stomach	Primary	Low	ANED	*KIT*	11	p.Val559Asp	missense	
30	77	Stomach	Primary	High	ANED	*KIT*	11	p.Val559Asp	missense	
31	69	Small intestine	Primary	High	ANED	*KIT*	11	p.Val559Ala	missense	
42	66	Stomach	Primary	Very low	ANED	*KIT*	11	p.Trp557Arg	missense	
46	72	Stomach	Primary	Very low	ANED	*KIT*	11	p.Val559Asp	missense	
54	66	Stomach	Primary	High	ANED	*KIT*	11	p.Val559Asp	missense	Yes
56	nd	Stomach	Primary	nd	ANED	*KIT*	11	p.Val559Asp	missense	
58a	61	Liver	Metastasis		AWD	*KIT*	11	p.Leu576Pro	missense	
59	75	Stomach	Primary	Very low	ANED	*KIT*	11	p.Val559Asp	missense	

Case ID	Age	Location	Sample type	Risk Group[1]	Status	Gene	Exon	Mutation description[2]	Type	CGH?
65	68	Colon	Recurrence		AWD	*KIT*	11	p.Val560Asp	missense	
75	76	Stomach	Primary	Moderate	ANED	*KIT*	11	p.Val559Ala	missense	
77	70	Small intestine	Primary	Very low	ANED	*KIT*	11	p.Trp557Arg	missense	
78	72	Small intestine	Primary	Moderate	ANED	*KIT*	11	p.Leu576Pro	missense	
79	56	Small intestine	Primary	High	AWD	*KIT*	11	p.Val560Glu	missense	
80	71	Peritoneum	Metastasis		AWD	*KIT*	11	p.Trp557Arg	missense	
5b	84	Peritoneum	Metastasis		DFD	*KIT*	13	p.Val654Glu	missense	
58b	61	Stomach	Recurrence		AWD	*KIT*	13	p.Val654Glu	missense	
14	63	Small intestine	Recurrence		DFD	*KIT*	17	p.Asp820Tyr	missense	Yes
40	57	Stomach	Primary	High	AWD	*KIT*	17	p.Asp820Tyr	missense	Yes
21	42	Small intestine	Primary	Low	ANED	*PDGFRA*	12	p.Ser566_Glu571delinsArg	delins	Yes
66	83	Stomach	Primary	Very low	ANED	*PDGFRA*	12	p.Val561Asp	missense	
22	53	Stomach	Primary	Low	ANED	*PDGFRA*	14	p.Asn569Tyr	missense	Yes
7	58	Stomach	Primary	Low	ANED	*PDGFRA*	18	p.Met844_Ser847del	deletion	Yes
17	44	Peritoneum	Primary	High	ANED	*PDGFRA*	18	p.Met844_Ser847del	deletion	
6	64	Stomach	Primary	Very low	ANED	*PDGFRA*	18	p.Asp842Val	missense	Yes
9	62	Stomach	Primary	Low	ANED	*PDGFRA*	18	p.Asp842Val	missense	Yes
50	61	Stomach	Primary	Low	ANED	*PDGFRA*	18	p.Asp842Val	missense	Yes
53	68	Stomach	Primary	Very low	ANED	*PDGFRA*	18	p.Asp842Val	missense	Yes

Case ID	Age	Location	Sample type	Risk Group[1]	Status	Gene	Exon	Mutation description[2]	Type	CGH?
1	80	Stomach	Primary	Very low	ANED	-	-	-	-	Yes
8	77	Colon	Primary	Moderate	ANED	-	-	-	-	Yes
25	46	Small intestine	Primary	High	DFD	-	-	-	-	
32	72	Stomach	Primary	Very low	DNED	-	-	-	-	Yes
38	67	Stomach	Primary	Moderate	ANED	-	-	-	-	
39	30	Peritoneum	Primary	High	ANED	-	-	-	-	
63	49	Stomach	Primary	High	ANED	-	-	-	-	Yes
64	68	Peritoneum	Primary	High	DWD	-	-	-	-	
67	55	Stomach	Primary	Very low	ANED	-	-	-	-	
76	45	Stomach	Primary	nd	nd	-	-	-	-	

[1]Risk stratification of primary tumors follows the NCCN Clinical Practice Guidelines for GIST (Demetri et al, 2007). [2]Mutant sequences at the protein level are deduced from the mutations identified at the DNA level. Mutation nomenclature follows the recommendations of the Human Genome Variation Society (http://www.hgvs.org). Abbreviations: ANED, alive, no evidence of disease; AWD, alive with disease; DNED, dead, no evidence of disease; DWD, dead with disease; DFD, dead from disease; nd, not determined.
