# Supplementary material for: Chromosome copy number changes carry prognostic information independent of KIT/PDGFRA point mutations in gastrointestinal stromal tumors
Source: BMC Med. 2010 May 14;8:26. doi: 10.1186/1741-7015-8-26 (PMC2876987; doi:10.1186/1741-7015-8-26)
Supplement: Additional file 2 — Chromosomal imbalances and molecular alterations detected in 29 GIST submitted to CGH analysis. Table integrating CGH and genotypic data for 29 patients for which both information was available. [file 1741-7015-8-26-S2.PDF]

**Supplementary Table 2: Chromosomal imbalances and molecular alterations detected in 29 GIST submitted to CGH analysis.**

| ID | Gene | Exon | Mutation* | CGH findings** |
|---|---|---|---|---|
| 2 | *KIT* | 9 | p.Ala502_Tyr503dup | rev ish dim(X)(p11p22),dim(1)(p13p36),enh(1)(q21),enh(1)(q32),dim(2)(q11q37),enh(3)(p21), enh(3)(q21),enh(3)(q28q29),enh(6)(p21),enh(7)(q22),enh(7)(q36),enh(8)(p11p23),enh(9)(p12p13), enh(9)(q22q34),enh(10)(q22q26),dim(11)(p14p15),enh(11)(q13q14),enh(12)(p13),enh(12)(q13), enh(12)(q22q24),dim(13)(q),dim(14)(q),dim(15)(q15q26),enh(16),enh(17),enh(19),enh(20)(q),dim(21)(q) |
| 18a | *KIT* | 9 | p.A502_Y503dup | rev ish dim(1)(p13p36),dim(2)(p25q13),dim(11),enh(12)(p12p13),dim(13)(q),dim(15)(q),enh(21)(q) |
| 55 | *KIT* | 9 | p.Ala502_Tyr503dup | rev ish dim(1)(p13p36),dim(6)(q12q27),dim(9)(p21p24),dim(14)(q),dim(18)(p11q21),dim(19)(p13), enh(20)(p12p13),enh(20)(q),dim(22)(q) |
| 3 | *KIT* | 11 | p.Trp557_Lys558del | rev ish enh(Y),dim(1)(p21p36),enh(1)(q21q23),enh(1)(q42q44),dim(2)(q11q37),enh(6)(p12p25), dim(6)(q24q27),enh(8)(p21p22),enh(12)(q21q24),dim(13)(q),dim(14)(q),dim(15)(q21q26), enh(17)(q11q24),dim(18)(q21q23),dim(22)(q) |
| 4 | *KIT* | 11 | p.Lys550_Gln556del | rev ish enh(6)(q26q27),dim(10),dim(14)(q),dim(15)(q),dim(22)(q) |
| 44 | *KIT* | 11 | p.Val559_Glu561del | rev ish dim(14)(q),dim(15)(q),dim(22)(q) |
| 60 | *KIT* | 11 | p.Asp579del | rev ish dim(14)(q) |
| 62 | *KIT* | 11 | p.Lys550_Lys558del | rev ish dim(11)(p),dim(14)(q),dim(22)(q) |
| 13 | *KIT* | 11 | p.Tyr553_Leu576delinsAsnCysLeuHis LeuTyrSerSerGln | rev ish dim(14)(q) |
| 34 | *KIT* | 11 | p.Trp557_Val559delinsPhe | rev ish dim(1)(p13p36),enh(1)(q21q44),dim(2)(p),enh(2)(q14q36),enh(3),enh(4),enh(5)(p12p15), enh(5)(q12q35),enh(6)(p12p25),dim(6)(q16q27),enh(7)(p13p22),enh(7)(q21q35),enh(8)(p22p23), enh(8)(q13q24),enh(10)(p13q22),enh(10)(q24),enh(11)(p14p15),dim(11)(q22q23), enh(12)(q14q22),dim(13)(q),dim(14)(q),dim(15)(q),dim(18)(q),dim(21)(q),dim(22)(q) |
| 45 | *KIT* | 11 | p.Lys558_Thr574delinsAsnArgSer | rev ish dim(1)(p12p36),dim(2)(p22p23),dim(3)(q12q22),dim(4)(p12p16),dim(7)(p),dim(9)(q22q32) |
| 10 | *KIT* | 11 | p.Asp572_His580dup | rev ish dim(X)(q21q28),dim(14)(q) |
| 72 | *KIT* | 11 | p.Pro585_Arg586insThrThr GlnLeuProTyrAspHisLysTrpGluPhePro | rev ish dim(14)(q),enh(20)(q) |
| 5a | *KIT* | 11 | p.Trp557Arg | rev ish dim(1)(p13p36),dim(3)(p21p25),dim(14)(q22q32),dim(15)(q12q22),enh(17)(q21q25),dim(22)(q) |
| 54 | *KIT* | 11 | p.Val559Asp | rev ish dim(14)(q),dim(22)(q) |
| 15b | *KIT* | 11 | p. Trp557Gly | rev ish dim(1)(p36q21),enh(10)(q22q23),dim(10)(q24q26),dim(14)(q),dim(15)(q21q26),dim(22)(q) |
| 14 | *KIT* | 17 | p.Asp820Tyr | rev ish dim(1)(p21p34),dim(3)(q12q26),dim(8)(p21p23),dim(11),enh(12),dim(22)(q) |
| 40 | *KIT* | 17 | p.Asp820Tyr | rev ish dim(14)(q),dim(15)(q) |
| 21 | *PDGFRA* | 12 | p.Ser566_Glu571delinsArg | rev ish dim(14)(q) |

| ID | Gene | Exon | Mutation* | CGH findings** |
|---|---|---|---|---|
| 22 | *PDGFRA* | 14 | p.Asn569Tyr | No copy number changes |
| 7 | *PDGFRA* | 18 | p.Met844_Ser847del | rev ish dim(12)(p),enh(12)(q12q24),dim(14)(q) |
| 6 | *PDGFRA* | 18 | p.Asp842Val | rev ish dim(14)(q),dim(22)(q) |
| 9 | *PDGFRA* | 18 | p.Asp842Val | rev ish dim(14)(q22q32) |
| 50 | *PDGFRA* | 18 | p.Asp842Val | rev ish enh(10)(q26),dim(14)(q) |
| 53 | *PDGFRA* | 18 | p.Asp842Val | rev ish dim(1)(p),enh(5),dim(10)(q21q26),dim(14)(q12q32) |
| 63 | - | - | - | rev ish dim(X),dim(1)(p12p36),enh(1)(q21q44),dim(3)(q13q29),dim(9)(q22q34),enh(11)(p15), enh(11)(q13q25),dim(13)(q),dim(14)(q) |
| 1 | - | - | - | No copy number changes |
| 8 | - | - | - | No copy number changes |
| 32 | - | - | - | No copy number changes |

*Mutant sequences at the protein level are deduced from the mutations identified at the DNA level. Mutation nomenclature follows the recommendations of the Human Genome Variation Society (http://www.hgvs.org). **CGH descriptions follow the guidelines proposed by the International System for Human Chromosome Nomenclature (ISCN) 2005. Abbreviations: delins, deletion insertion mutation; rev, reverse; ish, in situ hybridization; dim, diminished; enh, enhanced.
