# Supplementary material for: Chromosome copy number changes carry prognostic information independent of KIT/PDGFRA point mutations in gastrointestinal stromal tumors
Source: BMC Med. 2010 May 14;8:26. doi: 10.1186/1741-7015-8-26 (PMC2876987; doi:10.1186/1741-7015-8-26)
Supplement: Additional file 3 — Disease-free survival results for selected variables. Univariate disease-free survival results for selected genetic and clinical variables, using the Kaplan-Meyer test with Log-rank statistics at 60 months follow-up. [file 1741-7015-8-26-S3.PDF]

Supplementary Table 3. Disease-free survival results for selected variables (univariate analysis, Kaplan-meyer test with Log-rank statistics at 60 months)

Variable	n	Mean Survival (months)	Time to 50% survival (months)	p-value
Risk groups				
Low/Very Low	27	58	-	
Moderate	8	53	-	
High	27	37	38	*0.001*
Genomic complexity				
<3 CNCs	15	56	-	
≥3 CNCs	12	28	24	*0.002*
Genomic Gains?				
no	13	56	-	
yes	14	31	24	*0.006*
Loss of 1p				
no	16	53	-	
yes	11	29	24	*0.009*
Loss of 22q				
no	16	53	-	
yes	11	29	21	*0.006*
Mutated gene				
KIT	49	nd		
PDGFRA	9	nd	nd	*0.039*
