# Supplementary material for: Chromosome copy number changes carry prognostic information independent of KIT/PDGFRA point mutations in gastrointestinal stromal tumors
Source: BMC Med. 2010 May 14;8:26. doi: 10.1186/1741-7015-8-26 (PMC2876987; doi:10.1186/1741-7015-8-26)
Supplement: Additional file 4 — Multivariate disease-free survival results. Results of the cox-regression models (forward conditional setting) using the variables that showed significant prognostic value in univariate testing. [file 1741-7015-8-26-S4.PDF]

Supplementary Table 4a. Multivariate disease-free survival results using risk groups and genotypic information (Cox-regression model, forward conditional setting)

Output Variable(s)	n	Hazard Ratio	95% CI for HR	p-value
Risk group				
Low/Moderate Vs.	35	-		
High	27	9.78	2.22 - 43.11	0.003

Inputed variables were "risk groups" and "genotype" (Wildtype vs. KIT vs. PDGFRA mutations), comprising 61 valid samples. For the purposes of this test, recurrences and metastases were included and coded in the High-Risk group. Abbreviations: CI, confidence interval; HR, hazard ratio.

Supplementary Table 4b. Multivariate disease-free survival results using risk groups, genotypic and CGH information (Cox-regression model, forward conditional setting)

Output Variable(s)	n	Hazard Ratio	95% CI for HR	p-value
Genomic complexity				
<3 CNCs Vs.	15	-		
≥3 CNCs	12	13.68	1.68 - 111.11	0.014

Inputed variables were "risk groups", "genomic complexity", "genotype", "genomic gains", "losses at 1p" and "losses at 22q", comprising 27 valid samples. Abbreviations: CI, confidence interval; HR, hazard ratio.
